# Analysis of diagnoses extracted from electronic health records in a large mental health case register

**DOI:** 10.1371/journal.pone.0171526

**Published:** 2017-02-16

**Authors:** Yevgeniya Kovalchuk, Robert Stewart, Matthew Broadbent, Tim J. P. Hubbard, Richard J. B. Dobson

**Affiliations:** 1 School of Computing and Digital Technology, Faculty of Computing, Engineering and the Built Environment, Birmingham City University, Birmingham, United Kingdom; 2 Department of Psychological Medicine, The Institute of Psychiatry, Psychology & Neuroscience, King’s College London, London, United Kingdom; 3 NIHR Biomedical Research Centre for Mental Health and Biomedical Research Unit for Dementia at South London and Maudsley NHS Foundation, London, United Kingdom; 4 Department of Medical & Molecular Genetics, Faculty of Life Sciences & Medicine, King’s College London, London, United Kingdom; 5 Department of Biostatistics & Health Informatics, The Institute of Psychiatry, Psychology & Neuroscience, King’s College London, London, United Kingdom; 6 Farr Institute of Health Informatics Research, London Institute of Health Informatics, University College London; and the NIHR University College London Hospitals Biomedical Research Centre, London, United Kingdom; Yokohama City University, JAPAN

## Abstract

The UK government has recently recognised the need to improve mental health services in the country. Electronic health records provide a rich source of patient data which could help policymakers to better understand needs of the service users. The main objective of this study is to unveil statistics of diagnoses recorded in the Case Register of the South London and Maudsley NHS Foundation Trust, one of the largest mental health providers in the UK and Europe serving a source population of over 1.2 million people residing in south London. Based on over 500,000 diagnoses recorded in ICD10 codes for a cohort of approximately 200,000 mental health patients, we established frequency rate of each diagnosis (the ratio of the number of patients for whom a diagnosis has ever been recorded to the number of patients in the entire population who have made contact with mental disorders). We also investigated differences in diagnoses prevalence between subgroups of patients stratified by gender and ethnicity. The most common diagnoses in the considered population were (recurrent) depression (ICD10 codes F32-33; 16.4% of patients), reaction to severe stress and adjustment disorders (F43; 7.1%), mental/behavioural disorders due to use of alcohol (F10; 6.9%), and schizophrenia (F20; 5.6%). We also found many diagnoses which were more likely to be recorded in patients of a certain gender or ethnicity. For example, mood (affective) disorders (F31-F39); neurotic, stress-related and somatoform disorders (F40-F48, except F42); and eating disorders (F50) were more likely to be found in records of female patients, while males were more likely to be diagnosed with mental/behavioural disorders due to psychoactive substance use (F10-F19). Furthermore, mental/behavioural disorders due to use of alcohol and opioids were more likely to be recorded in patients of white ethnicity, and disorders due to use of cannabinoids in those of black ethnicity.

## Introduction

In 2014, the Department of Health in England issued a report acknowledging that “for decades the health and care system in England has been stacked against mental health services” with the distribution of resources favouring only physical health services [1]. More funding was promised to improve mental health services [1–3] to ensure that mental and physical health conditions are treated equally [1, 4]. Decisions on allocating funding are frequently based on surveys and reports compiled by specialist groups [5–7] and charities [8]. Electronic healthcare records (EHRs) are another potentially rich resource of patient data, and analysis of such data can reveal patterns and trends in healthcare provision, patients’ profiles and their health problems. While a lot of effort still needs to be invested to integrate separate EHRs systems in order to generate a more complete picture of patients’ pathways [9–11], researchers and clinicians should make the most of existing systems owned by separate hospitals and NHS trusts.

In this paper, we analyse data from a database which contains information from service users at one of the largest mental health providers in Europe, the South London and Maudsley NHS Foundation Trust (SLaM) [12]. SLaM serves a geographic catchment of over 1.2 million residents in four south London boroughs (Croydon, Lambeth, Lewisham and Southwark), and its EHR database includes patients’ demographic details, symptoms, diagnoses, test scores, medications prescribed, and records of clinical events (referrals, admissions, discharges, etc.). In order to facilitate research, a de-identified version of the SLaM EHR called the Clinical Record Interactive Search (CRIS) system [13] was developed in 2008.

The majority of information in the database is stored in the form of free text, including correspondence and narratives recorded by clinicians during healthcare encounters. In this study however, we focus on semi-structured fields, which contain patients’ diagnoses recorded as ICD10 codes [14]. This analysis sought to provide a benchmark to which the information we plan to mine from free text can be compared.

CRIS data have supported a range of research projects [15–21]. However, these studies have concentrated on developing tools or answering specific clinical or research questions. The aim of this paper is to present descriptive statistics of diagnoses recorded in the database. In particular, we report prevalence of the most common diagnoses in the entire patient population and in subgroups stratified by gender and ethnicity. This research is an updated and extended analysis of an earlier report [12]. More specifically, unlike the previous study which reports statistics based on primary diagnoses of active population only, this paper considers both primary and secondary diagnoses recorded for the entire population of patients accepted by SLaM up until May 2015, takes into account patients’ gender and ethnicity, as well as provides results on a more detailed level of ICD10 code hierarchy (we did not seek to take into consideration age of patients as age at first time episodes is currently not readily available in the database).

Looking into differences in health problems experienced by people with certain demographic characteristics may help to understand individual needs of patients and root causes of their mental health problems. It is suggested, for example, that “there are ethnic as well as socioeconomic dimensions to the prevalence of mental ill-health” [22] and that “different ethnic groups have different rates and experiences of mental health problems, reflecting their different cultural and socio-economic contexts and access to culturally appropriate treatments” [23]. For instance, according to a survey of black and minority ethnic people experiencing mental health difficulties (conducted during February to March 2013 in England) [24], Asians experienced more depression and anxiety than black groups, while more black people than Asians were diagnosed with schizophrenia.

There are also gender-specific differences in prevalence of mental health disorders. In 2001 and 2003 for example, the Office for National Statistics reported that women were more likely to have been treated for a mental health problem than men (29% compared to 17%), with depression and anxiety being more prevalent in women, while alcohol or drug problems–in men [25, 26]. According to more recent surveys, the prevalence of autism is higher in men than women [27], while eating disorders are more common among women than men [28, 29].

Statistics provided in surveys are usually either general (reporting on all or several disorders combined) or focusing on a few specific disorders. Moreover, these statistics are likely to change over time, especially in recent years when more people become more aware of mental wellbeing [22, 30] and are more likely to step forward reporting potential problems. It is therefore important to regularly monitor changes in the usage of mental health services. With this paper, we aim to capture the current statistics for a broad spectrum of mental health issues, against which future shifts in diagnoses distribution across patient subgroups could be studied. Furthermore, we compare the statistics rendered from our EHR with other sources highlighting commonalities, differences and additional information not reported previously.

## Data and methods

A de-identified version of the SLaM EHR called the Clinical Record Interactive Search (CRIS) system [13] was used as a data source for this study. Ethical approval as an anonymised database for secondary analysis was originally granted in 2008, and renewed for a further 5 years in 2013 (Oxford C Research Ethics Committee, reference 08/H0606/71+5). The study presented in this paper has been approved by the CRIS Oversight Committee [13].

For our analysis, we assembled a subset of records on 203,427 patients registered in the CRIS database between November 2008 and May 2015: 101,549 males and 101,813 females (65 with gender not recorded). Overall, there were 562,726 primary and secondary diagnoses recorded in structured fields for these patients, employing 2,531 unique ICD10 codes. We noted however, that not all diagnoses were recorded at their lowest (most specific) level of hierarchy, so as well as ‘F20.0—paranoid schizophrenia’ there are also cases of ‘F20 –Schizophrenia’, for example. To address this issue, we trimmed each code to its decimal point (i.e. taking only its letter and the following two digits). Since several diagnosis codes could be recorded for a patient, and the same diagnosis may be recorded several times on different dates for the same patient, we calculated overall and unique case counts for each code.

In this paper, we explore how unique diagnoses recorded for at least 100 unique patients were distributed across different genders and ethnicities and if there were any significant differences in their prevalence. We performed two statistical analyses, one comparing genders, and a second comparing ethnic groups. In both cases, we took the same cohort of 203,427 patients, but had to remove 65 patients from the gender analysis where no gender was recorded, and 29,559 patients from the ethnicity analysis where ethnicity was absent. While a detailed ethnic category was specified for each patient, we have aggregated them into four ethnic groups. The ‘White’ ethnic group includes ‘British’, ‘Irish’, and ‘Any other white background’ ethnic categories. The ‘Black’ group includes ‘African’, ‘Caribbean’, and ‘Any other black background’ categories. The ‘Asian’ group refers to ‘Bangladeshi’, ‘Chinese’, ‘Indian’, ‘Pakistani’, and ‘Any other Asian background’. The ‘Other’ ethnicity group includes patients with mixed backgrounds, such as ‘White and Asian’, ‘White and Black African’, ‘White and Black Caribbean’ and ‘Any other mixed backgrounds’.

To test statistical significance of diagnostic enrichment for a given gender and ethnic group, we calculated p-values for each diagnostic code generated from Chi-square scores. Since multiple comparisons were involved in the testing (110 codes for 2 and 4 categories of gender and ethnicity respectively), we also calculated q-values by adjusting each p-value using the False Discovery Rate Benjamini-Hochberg method [31]. We performed this analysis at two different levels of the ICD10 code hierarchy: the third level (codes trimmed to letter and the following two digits) and the highest level (trimmed to include letter only). The first analysis informs about differences in the population across various mental health condition, while the second shows differences across the codes that belong to chapters other than ‘V—Mental and Behavioural Disorders’.

## Results

Overall, 36.7% of diagnoses made for all patients were repeated diagnoses, with average repetition rate of 16.9% per code (st. dev. = 19.2). Of all diagnoses, 14.3% (or 16.3% of unique records per patient) were ‘F99—Unspecified mental disorder’, and 10.1% (or 12.1% of unique records per patient) were ‘Z71.1—Person with feared complaint in whom no diagnosis is made’, resulting in 46.0% of patients who had at least one of either F99 or Z71.1 code assigned, only about half of whom (23.6% of patients) had another (more specific) code recorded alongside; 22.4% of all patients did not have any other defined diagnosis recorded.

Following the non-specific F99 and Z71.1 codes, the most common diagnoses were depressive episode (recorded in 13.2% of patients), reaction to severe stress and adjustment disorders (7.1%) and mental and behavioural disorders due to use of alcohol (6.9%). Table 1 includes the top 10 most frequent (defined) diagnoses along with their unique and overall case counts, and percentages of the patients for whom the diagnoses were made. It is worth noting that some patients had a record of recurrent depressive episode (F33 code) following the diagnosis of depressive episode (F32) made on an earlier date. Combining the two diagnoses resulted in 16.4% of patients who had either ‘depressive episode’ (F32 code) or ‘recurrent depressive episode’ (F33 code) recorded. See S1 Appendix for a complete list of frequency and repetition rates for each diagnosis; the list is ordered by descending unique case count.

**Table 1:** Top 10 most frequent ICD10 codes.

Code	Unique cases	Overall cases	% of patients
F32- Depressive episode	26759	40786	13.2
F43- Reaction to severe stress, and adjustment disorders	14414	20712	7.1
F10- Mental and behavioural disorders due to use of alcohol	14013	29545	6.9
F20- Schizophrenia	11403	37688	5.6
F41- Other anxiety disorders	9601	13867	4.7
F33- Recurrent depressive disorder	8973	15432	4.4
F00- Dementia in Alzheimer’s disease	8373	13363	4.1
F11- Mental and behavioural disorders due to use of opioids	7416	15634	3.7
F90- Hyperkinetic disorders	7258	9979	3.6
F84- Pervasive developmental disorders	6811	11224	3.4

**Fig 1:**
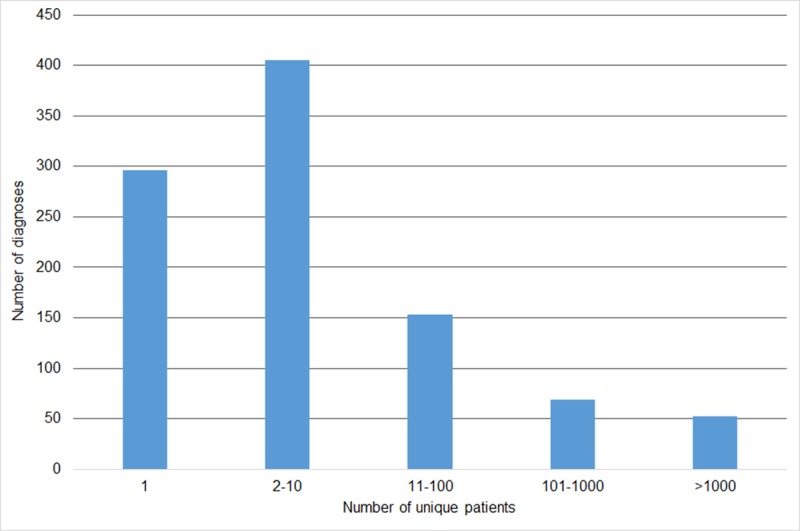
Number of diagnoses in each of the unique patient count bins. A bar chart showing the number of diagnoses that were assigned to each of the considered number of patients: 1 patient; between 2 and 10; 11 and 100; 101 and 1000; more than 1000 patients.

We found that many diagnosis codes were assigned to just one or only a few patients. When we grouped patient counts (1 patient, 2 to 20 patients, 11 to 100 patients, 101–1000 patients, and over 1000 patients) and calculated the number of unique diagnoses made per number of unique patients in each of these groups, we found that only 53 diagnoses (5.4%) were assigned to more than a thousand unique patients (Fig 1).

To study the difference of diagnoses prevalence across different genders and ethnicities, we only took diagnoses recorded for at least 100 unique patients (i.e. the last two groups on the right of Fig 1). Table 2 contains the number of SLaM patients in each of the gender and ethnicity group and proportion these counts make of the total respective (gender or ethnicity) cohort. The table also presents percentages of residents of different gender and ethnicity in the SLaM catchment area, London and England as a whole, out of the entire population in the respective areas, derived from the 2011 UK Census [32]. Note, there are approximately the same number of males and females in the database, while representation of different ethnicities varies, with white patients being in majority and Asians in minority.

**Table 2:** Patient counts and true population rates in subgroups stratified by gender and ethnicity.

	Female	Male		White	Black	Asian	Other
**SLaM patient count**	101813	101549		114501	33388	8568	17411
**% of total patient cohort**	50.1	49.9		56.3	16.4	4.2	8.6
**SLaM catchment (% of its true total population)**	50.9	49.1		55.1	24.7	10.8	9.4
**London (% of its true total population)**	50.7	49.3		59.8	13.3	18.4	8.5
**England (% of its true total population)**	50.8	49.2		85.5	3.4	7.7	3.4

Table 3 summarises the findings highlighting the differences in codes related to mental health which have q-values below 0.01 in either gender or ethnicity testing, or both (results are provided in alphabetic order of the codes). Full results of gender and ethnicity enrichment analyses can be found in S2 Appendix, where for each diagnostic code we show numbers in diagnostic groups, Chi-square scores, p- and q-values. Results in S2 Appendix are provided in ascending order of p-values separately for gender and ethnicity, and at two levels of ICD10 codes hierarchy.

**Table 3:** Prevalence of diagnoses (listed by ICD10 code) in subgroups of patients stratified by gender and ethnicity. M-Male; F-Female; W-White; B-Black; A-Asian; O-Other ethnicities; ‘-’—no significant difference. Shown gender is associated with higher recorded rates per diagnosis. Ethnicities mentioned in capitals have relative count for the diagnosis above the average across all 4 ethnicities, while lower case letters represent ethnicities with relative count below the average.

Code	Name	Gender			Ethnicity		
		M,F,-	Chi-Square (1 df)	q-value	w,b,a,o,-	Chi-Square (3 df)	q-value
F00	Dementia in Alzheimer disease	F	956.518	<0.001	WbAo	888.780	<0.001
F01	Vascular dementia	F	82.280	<0.001	WBao	266.434	<0.001
F02	Dementia in other diseases classified elsewhere (mostly F02.3—Dementia in Parkinson disease)	M	36.350	<0.001	WBAo	52.270	<0.001
F03	Unspecified dementia	F	203.309	<0.001	WBao	326.040	<0.001
F05	Delirium, not induced by alcohol and other psychoactive substances	F	39.340	<0.001	Wbao	376.484	<0.001
F06	Other mental disorders due to brain damage and dysfunction and to physical disease	-	0.172	0.718	WBAo	135.148	<0.001
F07	Personality and behavioural disorders due to brain disease, damage and dysfunction	M	53.959	<0.001	WbAo	15.175	0.002
F10	Mental and behavioural disorders (MBD) due to use of alcohol	M	1833.336	<0.001	Wbao	1505.215	<0.001
F11	MBD due to use of opioids	M	1403.687	<0.001	Wbao	804.068	<0.001
F12	MBD due to use of cannabinoids	M	630.262	<0.001	wBao	263.450	<0.001
F13	MBD due to use of sedatives or hypnotics	M	49.128	<0.001	Wbao	181.671	<0.001
F14	MBD due to use of cocaine	M	328.713	<0.001	WBao	84.436	<0.001
F15	MBD due to use of other stimulants, including caffeine	M	84.827	<0.001	WBaO	14.020	0.004
F16	MBD due to use of hallucinogens	M	15.680	<0.001	-	8.112	0.053
F19	MBD due to multiple drug use and use of other psychoactive substances	M	1298.514	<0.001	WBao	99.786	<0.001
F20	Schizophrenia	M	753.058	<0.001	wBAo	4003.993	<0.001
F22	Persistent delusional disorders	-	7.257	0.010	wBAo	226.666	<0.001
F23	Acute and transient psychotic disorders	M	16.736	<0.001	wBAo	1618.696	<0.001
F25	Schizoaffective disorders	-	4.270	0.052	wBao	916.484	<0.001
F28	Other nonorganic psychotic disorders	-	0.195	0.710	wBAo	188.240	<0.001
F29	Unspecified nonorganic psychosis	M	69.873	<0.001	wBao	940.170	<0.001
F30	Manic episode	-	<0.001	1	wBAo	108.127	<0.001
F31	Bipolar affective disorder	F	180.123	<0.001	wBAo	15.214	0.002
F32	Depressive episode	F	1629.451	<0.001	wbAO	146.089	<0.001
F33	Recurrent depressive disorder	F	789.744	<0.001	WbaO	183.675	<0.001
F34	Persistent mood [affective] disorders	F	91.788	<0.001	WbaO	58.520	<0.001
F38	Other mood [affective] disorders	F	68.813	<0.001	-	5.644	0.146
F39	Unspecified mood [affective] disorder	F	68.120	<0.001	-	11.804	0.010
F40	Phobic anxiety disorders	F	57.481	<0.001	WbaO	119.419	<0.001
F41	Other anxiety disorders	F	357.590	<0.001	WbaO	528.133	<0.001
F42	Obsessive-compulsive disorder	-	0.006	0.956	WbAO	399.400	<0.001
F43	Reaction to severe stress, adjustment disorders	F	236.409	<0.001	wBAO	600.891	<0.001
F44	Dissociative [conversion] disorders	F	233.983	<0.001	Wbao	49.464	<0.001
F45	Somatoform disorders	F	118.929	<0.001	WbAO	67.779	<0.001
F48	Other neurotic disorders	F	227.153	<0.001	Wbao	246.767	<0.001
F50	Eating disorders	F	3549.500	<0.001	WbaO	763.240	<0.001
F52	Sexual dysfunction, not caused by organic dis.	M	254.526	<0.001	WbAo	32.379	<0.001
F60	Specific personality disorders	F	240.954	<0.001	WbaO	187.197	<0.001
F61	Mixed and other personality disorders	M	24.641	<0.001	WbaO	25.092	<0.001
F64	Gender identity disorders	M	20.180	<0.001	wbaO	61.176	<0.001
F70	Mild mental retardation	M	108.092	<0.001	wBao	38.853	<0.001
F71	Moderate mental retardation	M	77.455	<0.001	wBao	64.696	<0.001
F72	Severe mental retardation	M	100.200	<0.001	wBAo	145.710	<0.001
F78	Other mental retardation	M	19.417	<0.001	WBao	26.483	<0.001
F79	Unspecified mental retardation	M	13.641	<0.001	wBao	69.221	<0.001
F80	Specific development dis of speech and language	M	261.996	<0.001	wBaO	175.098	<0.001
F81	Specific developmental dis of scholastic skills	M	159.399	<0.001	WBaO	14.130	0.004
F82	Specific developmental disorder of motor function	M	56.457	<0.001	-	3.061	0.382
F83	Mixed specific developmental disorders	M	26.674	<0.001	wBaO	33.551	<0.001
F84	Pervasive developmental disorders	M	2354.401	<0.001	wBao	48.657	<0.001
F89	Unspecified dis of psychological development	M	25.135	<0.001	wBaO	51.500	<0.001
F90	Hyperkinetic disorders	M	2292.562	<0.001	WbaO	186.085	<0.001
F91	Conduct disorders	M	581.926	<0.001	wBaO	264.079	<0.001
F92	Mixed disorders of conduct and emotions	M	130.335	<0.001	wBaO	491.193	<0.001
F93	Emotional dis with onset specific to childhood	F	22.375	<0.001	wBaO	210.708	<0.001
F94	Disorders of social functioning with onset specific to childhood and adolescence	M	14.295	<0.001	wBaO	42.653	<0.001
F95	Tic disorders	M	100.754	<0.001	WbaO	28.918	<0.001
F98	Other behavioural and emotional dis with onset usually occurring in childhood and adolescence	M	67.050	<0.001	wBaO	327.208	<0.001
G20	Parkinson disease	M	22.851	<0.001	WbAo	37.211	<0.001
R53	Malaise and fatigue	F	215.564	<0.001	Wbao	208.310	<0.001
S06	Intracranial injury	M		<0.001	-	3.726	0.309
X60	Intentional self-poisoning by nonopioid analgesics, antipyretics and antirheumatics	F	146.905	<0.001	wBaO	20.379	<0.001
X78	Intentional self-harm by sharp object	F	203.422	<0.001	wbaO	15.016	0.002
Z59	Problems related to housing and economic circumstances	M	17.404	<0.001	-	10.383	0.019
Z61	Problems related to negative life events in childhood	-	3.310	0.090	wBaO	85.581	<0.001
Z62	Other problems related to upbringing	-	2.969	0.109	wBao	27.235	<0.001
Z63	Other problems related to primary support group, incl. family circumstances	-	3.777	0.069	wBaO	33.757	<0.001
Z64	Problems rel. to certain psychosocial circumstances	-	0.022	0.907	wBao	16.985	0.001
Z65	Problems rel. to other psychosocial circumstances	-	0.492	0.553	wBao	17.501	<0.001
Z72	Problems related to lifestyle; (mostly Z72.2 drug use, Z72.1 alcohol use)	M	543.598	<0.001	WBao	104.018	<0.001

Women using mental health services were more likely than men to have received a diagnosis of mood (affective), neurotic, stress-related or eating disorder, while a diagnosis of mental or behaviour disorder due to substance use was more common in male service users. Most disorders causing dementia were recorded more often in female patients apart from dementia in Parkinson’s disease which was more common in male patients. Male patients were also more likely to have received diagnoses of schizophrenia, mental retardation, developmental disorders of speech and motor function, autism, conduct and hyperkinetic disorders, personality disorders due to brain disease, damage or dysfunction, and intracranial injury. The Z59 code (problems related to housing and economic circumstances) was recorded more often in males, while self-harm related (X) codes were more common in females.

Diagnoses of schizophrenia, schizotypal, delusional disorders and manic episodes were recorded more frequently for patients of black and Asian ethnicities compared to those of white and other ethnicities. Patients of black ethnicity were more likely to have a record of problems related to childhood, upbringing, social environment and psychosocial circumstances (Z60s codes), while patients of other (mixed) ethnicities were more likely to have received diagnoses of gender identity disorders, mixed and other personality disorders, and intentional self-harm. Substance use disorders involving alcohol, opioids and sedatives/hypnotics were more common among patients of white ethnicities, those involving cannabinoids were more common in black groups, and cocaine-related disorders were more common in both black and white ethnicities compared to other groups.

## Discussion

South London and Maudsley NHS Foundation Trust (SLaM) is one of the largest mental health providers in Europe serving a geographic catchment of over 1.2 million residents in four south London boroughs: Croydon, Lambeth, Lewisham and Southwark. In this paper, we analysed over 500,000 diagnoses recorded before June 2015 in the SLaM database for a population of approximately 200,000 patients who have been referred to SLaM with mental disorders.

According to our analysis, the largest group of patients (22.4%) did not have any defined diagnosis recorded, but were assigned with non-specific diagnosis codes (F99 and/or Z71.1) only. One factor contributing to this finding is the pressure on mental health services to have a diagnosis recorded on all people receiving care. This means that non-specific codes tend to get applied initially, during the period when patients are being assessed and before a specific diagnosis is concluded, which may represent a high proportion of patients’ time with the service. During the assessment phase, some patients may drop out and never receive a diagnosis; others may be not found to have a defined disorder. When a specific diagnosis is established, a treatment plan can be initiated and the patient can be discharged back to their primary care doctor (GP) with instructions. In such cases, there is a risk of clinicians making a diagnosis but not altering the diagnosis code in the database. One way around this administrative issue, is to perform text mining over unstructured clinicians’ notes to extract specific diagnoses, something we plan to do in the future. Text mining can also be useful to address the issue of many diagnosis codes not identifying meaningful patient groups (we established only 53 diagnoses, 5.4% of all, that are applied to groups of more than 1000 patients) and in cases when healthcare specialties find that most of the patients they see do not have one of the diagnoses determining the specialty (i.e., have ‘medically unexplained symptoms’).

In addition to the cases discussed above, many people with mental disorders do not receive secondary mental healthcare, so the patients represented in the SLaM database are a subset of everyone with mental disorders. This means that our findings cannot be directly compared to results presented in population based surveys and the intension of the following discussion is to demonstrate how our rates of defined diagnoses relate to the true population rates reported by others. Note also that our data do not capture potential differences in pathway to care that may affect different gender and minority groups. For example, a higher prevalence in one group compared to another might be because the first group have a higher risk of the disorder, or it might be that they have the same risk but the people in the first group are more likely to access mental healthcare (and therefore appear in the SLaM database).

We established that the most common diagnoses in the considered population were (recurrent) depression (ICD10 codes F32-33; 16.4% of patients), reaction to severe stress and adjustment disorders (F43; 7.1%), mental/behavioural disorders due to use of alcohol (F10; 6.9%), and schizophrenia (F20; 5.6%). We also found a substantial number of diagnoses that are more likely to be found in patients of a certain gender or ethnicity (q-values < 0.01). For example, our results support findings from previous surveys showing autism and problems related to alcohol and drugs being more prevalent in men [25–27], while depression, anxiety and eating disorders are more likely to be experienced by women [25, 26, 28, 29].

Consistently with the Dementia UK 2007 report [33], we found that dementia in Alzheimer’s disease is more common in women, while dementia in Parkinson’s disease is more prevalent in men. Our analysis does not support the reported statistics for vascular dementia; in our service, the diagnosis was recorded more often in women than men. However, it should be noted that gender ratios for dementia vary across age groups [33]. In particular, early onset dementia is higher in men than in women aged 50–65, while late onset dementia is marginally more common in women than in men (which could be related to longer life span on average in women).

Research suggests that the gender ratio relating to occurrence of deliberate self-harm changes with age [34]. Across all age groups however, our study supports the often reported statistics that self-harm related diagnoses are more prevalent among female patients [35].

Consistent with the earlier survey of ethnic minorities [24], we found that more people of Asian background were diagnosed with depression (ICD10 codes F32 and F33) and some anxiety disorders (F41 codes) compared to the black minority group. However, we found no difference between the two groups for phobic anxiety disorders (F40 codes).

As a further insight into substance use disorders, we found that those involving alcohol, opioids and sedatives/hypnotics were more common among patients of white ethnicities, those involving cannabinoids were more common in black groups, and cocaine-related disorders were more common in both black and white ethnicities than other groups.

In our analysis we included diagnostic codes related to physical health as they were recorded in our database (see S1 and S2 Appendices). Some of these codes have q-values below 0.01 (e.g., HIV, diabetes, asthma, hypertension, diseases of liver etc.). However, one should be careful interpreting these results as information related to physical health is likely to be recorded inconsistently by a mental health service provider.

## Conclusion and future work

In this paper, we reported frequencies of different diagnoses in the entire population of patients from the South London and Maudsley NHS Foundation Trust (see S1 Appendix) and explored prevalence of diagnoses (recorded for at least 100 patients) in subgroups of patients stratified by gender and ethnicity (see S2 Appendix).

Unfortunately, valid dates of diagnoses and encounters with mental health services, as well as age at first time episodes are not always available in our records; significant additional work is required to allow for any temporal analysis. Once we have addressed this issue, we will look into differences in diagnosis prevalence across subgroups of patients stratified by age, as well as analyse time-series of diagnoses. We also plan to employ additional information mined from free text and other relevant linked datasets, in order, for example, to obtain a more accurate picture of physical health of patients with mental health problems.

Research on the anonymised patient records data in the Case Register of the South London and Maudsley NHS Foundation Trust can be carried out subject to a collaborative agreement which adheres to strict patient-led governance.

## Supporting information

S1 Appendix Diagnosis frequencies. (XLSX)

S2 Appendix Chi-square scores, p- and q-values of diagnoses tested on enrichment for gender and ethnicity. (XLSX)
